# Influence of Reduced Graphene Oxide and Carbon Nanotubes on the Structural, Electrical, and Photoluminescent Properties of Chitosan Films

**DOI:** 10.3390/polym16131827

**Published:** 2024-06-27

**Authors:** Jesús R. González-Martínez, Ana B. López-Oyama, Deyanira Del Ángel-López, Crescencio García-Guendulain, Eugenio Rodríguez-González, Eder U. Pulido-Barragan, Felipe Barffuson-Domínguez, Aurora G. Magallanes-Vallejo, Pablo J. Mogica-Cantú

**Affiliations:** 1 Departamento de Investigación en Física (DIFUS), Universidad de Sonora, Blvd. Transversal S/N., Hermosillo 83000, Sonora, Mexico; jrgm18@hotmail.com; 2 Centro de Investigación en Ciencia Aplicada y Tecnología Avanzada, Unidad Altamira del Instituto Politécnico Nacional, Km. 14.5 Carr. Puerto Industrial, Altamira 89600, Tamaulipas, Mexico; ddelangel@ipn.mx (D.D.Á.-L.); eugenior62@gmail.com (E.R.-G.); eder.pulido.bar@gmail.com (E.U.P.-B.); aumagallanes@gmail.com (A.G.M.-V.); pmogicac2300@alumno.ipn.mx (P.J.M.-C.); 3 Conahcyt-Cicata Unidad Altamira, IPN. Km. 14.5 Carretera Puerto Industrial, Altamira 89600, Tamaulipas, Mexico; 4 Tecnologico de Monterrey, Escuela de Ingeniería y Ciencias, Blvd. Petrocel Km. 1.3, Altamira 89603, Tamaulipas, Mexico; 5 Departamento de Física, Universidad de Sonora, Blvd. Transversal S/N., Hermosillo 83000, Sonora, Mexico; felipe.barffuson@unison.mx

**Keywords:** carbon nanotubes, reduced graphene oxide, chitosan, film-forming properties, electrical properties, photoluminescence

## Abstract

Chitosan is a biopolymer with unique properties that have attracted considerable attention in various scientific fields in recent decades. Although chitosan is known for its poor electrical and mechanical properties, there is interest in producing chitosan-based materials reinforced with carbon-based materials to impart exceptional properties such as high electrical conductivity and high Young’s modulus. This study describes the synergistic effect of carbon-based materials, such as reduced graphene oxide and carbon nanotubes, in improving the electrical, optical, and mechanical properties of chitosan-based films. Our findings demonstrate that the incorporation of reduced graphene oxide influences the crystallinity of chitosan, which considerably impacts the mechanical properties of the films. However, the incorporation of a reduced graphene oxide–carbon nanotube complex not only significantly improves the mechanical properties but also significantly improves the optical and electrical properties, as was demonstrated from the photoluminescence studies and resistivity measurements employing the four-probe technique. This is a promising prospect for the synthesis of new materials, such as biopolymer films, with potential applications in optical, electrical, and biomedical bioengineering applications.

## 1. Introduction

Chitosan (Cs) is a naturally occurring material produced from chitin, usually found in crustaceans. Cs is a polysaccharide and, after cellulose, is the second largest renewable biopolymer. It is constituted of β-(1-4)-D-glucosamine units and β-(1-4)-N-acetyl-glucosamine according to the deacetylation degree of chitin. The chemical and functional properties of chitosan allow it to form gels and films, opening up new possibilities for industrial, optical, electrical, and biomedical applications. In recent years, chitosan films have attracted wide attention and have been applied in many fields, such as water treatment, dye removal, tissue engineering, wound healing [1,2], photocatalytic activity [3], and flexible electronics. Despite the numerous advantages and exceptional properties of Cs, its poor intrinsic mechanical and electrical properties impede its use in a wider range of applications. An effective approach for improving chitosan’s physical and mechanical properties is to develop films through the incorporation of fillers, such as clays, metal nanoparticles, carbon nanotubes, and/or reduced graphene oxide.

Dinakaran et al. [4] developed a composite nanostructure of nickel oxide (NiO) and NiO-embedded graphene hybrid nanostructure loaded into a PVDF (polyvinylidene difluoride) matrix. The nanocomposites were synthesized by hydrothermal reactions at 80 °C and fabricated through the solvent-casting technique. Buinov et al. fabricated conductive tissue engineering nanocomposite films based on chitosan and surfactant-stabilized graphene dispersions obtained by solution casting. Conductivity was linearly enhanced to 0.43 S/cm by increasing the concentration of rGO to 5 wt% [5]. Talebi et al. [6] prepared a polycaprolactone/chitosan/polypyrrole conductive composite film and reported that polypyrrole addition resulted in enhanced electrical conductivity. Although Cs has been widely used in many fields, there is scarce information on studying its electrical properties by embedding reduced graphene oxide and/or CNTs (carbon nanotubes). Investigations on the properties of nanocellulose [7,8,9,10,11], photoluminescent materials such as boron nitride [12], as well as carbon-based materials such as CNTs [13] and reduced graphene oxide (rGO) [14,15] have been developed for understanding the interesting properties of the distinct materials, which, when embedded in Cs, can integrate their individual properties for an enhanced synergistic effect in the development of electrically conductive films.

Reduced graphene oxide, a two-dimensional honeycomb lattice material, has attracted considerable attention due to its exceptional mechanical, thermal, electronic, optical, and electrical properties. Furthermore, rGO exhibits a large surface area, oxygen-containing chemical groups, strong ion exchangeability, and high electrical conductivity. These characteristics provide rGO/polymer composites with high strength, high modulus, and superior electrical conductivity. Barra et al. employed a green method for the preparation of electrically conductive bionanocomposites from chitosan and reduced graphene oxide subjected to a hydrothermal reduction using caffeic acid and then dispersed in chitosan. In the final bionanocomposites, the electrical conductivity was achieved at 0.7 S/m. Since increased tensile strength was accompanied by decreased water solubility, the reduced graphene oxide led to a significant improvement in the mechanical performance of the chitosan matrix [16].

Reduced graphene oxide–carbon nanotubes (rGO-CNTs) nanocomposites have been developed for enhancing electrochemical energy storage [17], supercapacitors [18,19], electromagnetic shielding [20], and water treatment [21]. Surprisingly, the development of chitosan-based films loaded with rGO-CNTs mixture has been employed only for a few applications, as shown below. Water-soluble rGO sheets grafted with chitosan effectively disperse multiwalled carbon nanotubes that efficiently enhance the well-known poor mechanical properties of Cs by the effective non-covalent π-π interactions between graphene sheets and CNTs through hydrogen bonding between grafted Cs were synthesized by Pan et al. [22], Mergen et al. [23] reported the study of electrical, optical, and mechanical properties of Cs/graphene nanoplatelets and Cs/MWCNT (multiwalled carbon nanotube) biocomposites films with promising properties for food packaging, UV protection, and biomedical applications. Their findings demonstrate that with the increase in graphene nanoplatelets or MWCNTs, the surface conductivity and mechanical and optical properties significantly increased.

In this paper, an rGO/Cs and rGO-CNTs/Cs composite film with enhanced electrical properties and photoluminescent properties was developed using the solvent-cast technique. When reduced graphene oxide was added, the mechanical properties of Cs films were improved, but not the electrical values. However, it is worth noting that the crystallinity of chitosan films was also improved. This indicates that rGO not only improves the mechanical properties of Cs but also improves the crystalline arrangement. In addition, we explore the synergistic effect of a mixture of rGO-CNTs into Cs. When the mixture was added to chitosan, the electrical conductivity was enhanced, demonstrating that CNTs considerably influence the carrier transport into Cs, improving the crystallinity, electrical conductivity, and photoluminescent (PL) properties of chitosan. This demonstrates that CNTs not only improve the mechanical properties of chitosan by physical enhancement but also enhance the electrical and optical properties of chitosan. Under the condition of maintaining high flexibility, the mechanical properties of chitosan films were improved as well, thus promoting the application of films in many fields, such as tissue engineering, biosensors, and biomedical applications.

## 2. Materials and Methods

### 2.1. Materials

Chitosan powder having a deacetylation degree of 75% (50 kDa, 190 kDa, and 310 kDa), multiwalled carbon nanotubes, HOPG (8000 mesh, purity 99.99%), HCl, NaCl, and acetic acid were acquired from Sigma-Aldrich, St. Louis, MO, USA. All the reagents were analytically graded and used as received without further purification. Reduced graphene oxide (rGO) was provided by the Center of Investigation in Applied Science and Advanced Technology, Altamira, México, previously synthesized as described in reference [14] and was employed without further purification.

### 2.2. Films Preparation

Films containing rGO and a combination of CNTs and rGO were made by dissolving chitosan of three molecular weights (low, medium, and high) in a 2.0% wt. aqueous solution of acetic acid. The solvent was then evaporated for 72 h at 30 °C under controlled conditions within a ventilated oven. Then, the films were characterized at room conditions of 35 °C and a relative humidity of ≈30%, characteristic of Hermosillo, Sonora, México city [coordinates 29°04′30″ N 110°57′30″ W].

For more details of labels assigned to the films produced in this work, see Supplementary Information (Figure S1).

### 2.3. UV–Vis Spectroscopy

The UV–visible spectra of the aqueous solutions were obtained using a PerkinElmer Lambda 45 dual-beam spectrophotometer at a scan rate of 280 nm/min. Measurements were made in the 200–900 nm range using a 1 cm quartz cell. Samples in acetic acid were allowed to equilibrate in the cell for one hour before being transferred to the quartz cell. The final volume was 3 mL.

### 2.4. Films Transparency

Following the procedure described by Vega et al. [24], the transparency of the film was calculated. Each sample was cut into a square shape measuring 1.25 × 1.25 cm and placed on the interior section of a spectrophotometer cell (Agilent Technologies Cary 5000 UV–vis NIR) that scanned within the 200–900 nm range. The measured absorbance at 800 nm (A_800_) was employed for transparency calculation, according to Han et al. [25].

(1) $$Transparency=\frac{A_{800}}{T}$$

A_800_ is the measure of absorbance at a wavelength of 800 nm, whereas T stands for the thickness of the film in millimeters. To obtain an average value, each film was measured three times.

### 2.5. Fourier Transform Infrared Spectroscopy

FTIR spectra were obtained by employing a Thermo Scientific FTIR instrument, the Nicolet 5 s model (Madison, WI, USA), equipped with an ATR iD3 accessory. A total of 64 scans were taken from 600 to 4000 cm^−1^ in range, with a resolution of 4 cm^−1^.

### 2.6. Films Thickness

We employed a digital micrometer (Mitutoyo, MDC-1 PX, Kawasaki-Shi, Kanawaga, Japan) to measure the film thickness. Four measurements were taken at different positions on the films [26].

### 2.7. Color Analysis

The film color analyses were conducted using a Minolta Chroma Meter colorimeter CR 300 (Osaka, Japan). The use of the CIELAB scale was employed in determining color parameters, specifically the lightness scale from black to white (L), and the chromaticity parameters, a* (redness–greenness) and b* (yellowness–blueness). All measures were carried out on a standard white plate. Six replicates for each sample were measured. The calculation of color differences (ΔE) was obtained as follows:

(2) $$∆E={\left[{\left(∆L^{*}\right)}^{2}+{\left(∆a^{*}\right)}^{2}+{\left(∆b^{*}\right)}^{2}\right]}^{1/2}$$

(3) $$∆L^{*}=L^{*}-L_{0},\;\;∆a=a^{*}-a_{0}\;\mathrm{and}\;{∆b}^{*}=b^{*}-b_{0}$$

$L^{*},\;{\;a}^{*},\;and\;b^{*},$ represent the standard color parameters while $L_{0},{\;a}_{0}$, and $b_{0}$ represent the color parameter of the sample.

### 2.8. Ionic Exchange Capacity (IEC)

We measured the IEC of three samples of each film using the titration method. The films were subjected to a prior IEC activation process, in which the films were alternately converted to the H^+^ and OH^−^ forms. The process involved exposing the films to a solution of 0.1 M HCl and 0.1 M NaCl for 48 h.

### 2.9. Swelling and Solubility

Swelling degree (DS) and solubility in water (WS) were analyzed. The state of equilibrium is reached by films (1.25 cm × 1.25 cm) immersed in distilled water at room temperature (25 °C) within an hour. The percentage of swelling degree (% DS) was determined through the following equation:

(4) $$\%DS=\frac{w_{e}-w_{0}}{w_{0}}$$

w_0_ is the initial dry weight of each film. In contrast, *w_e_* represents the weight of the absorbed films. After 24 h, the swollen films were dried at 60 °C. The percentage of solubility in water (*%SW*) is calculated:

(5) $$\%SW=\frac{w_{0}-w_{d}}{w_{0}}\times 100$$

where w_0_ is the initial weight of the dry films and w_d_ is the dry weight of the films after drying. The DS and SW values were measured in triplicate.

### 2.10. XRD Analysis

A Bruker D8 Advance X-ray diffractometer using Cu Ka radiation was used to obtain the X-ray diffraction patterns of the films. The X-ray wavelength was 0.15406 nm, and data were collected at a scan rate of 2°/min over a scattering angle range (2ϴ) of 5 to 90°.

### 2.11. Mechanical Characterization

A total of six specimens were tested for tensile strength (TS), elongation at break (EB), and Young’s modulus using a Texture Technologies Corp. texturometer under ASTM D882 [27]. The test was conducted at room temperature and ambient conditions (35 °C and ≈30% relative humidity). Each strip in all samples (10 mm wide, 40 mm long, 23 mm longitudinal slit) was cut with a razor blade. A crosshead speed of 10 mm/min and a gauge length of 30 mm were used as experimental parameters. Each film was subjected to five replicates of the tensile test.

### 2.12. X-ray Photoelectron Spectroscopy

The X-ray photoelectron spectroscopy (XPS) technique (PHI 5000 Versaprobe instrument, MN, USA) was utilized to conduct a surface analysis of four films selected for their use of MCs (medium molecular weight) chitosan (190 kDa) and the addition of carbon nanotubes and reduced graphene oxide. The high-resolution spectra for the detected and analyzed elements were obtained through the utilization of a monochromatic Mg Ka radiation as an X-ray source.

### 2.13. SEM Analysis

The high-magnification SEM images of medium-molecular-weight chitosan with rGO and rGO-CNTs were acquired using a JEOL JSM-7800F (Tokyo, Japan) with an operating energy of 5.0 eV.

### 2.14. Resistivity Measurements

The resistivity measurements of the films were carried out in ambient conditions using a source measuring unit (Keithley Model 4200-SCS system, Solon, OH, USA) equipped with a 0.40-inch equidistant tips configuration. The data were collected for 10 s with a gate voltage in the range of −0.0001 to +0.0001 V. The resistivity (ρ) measurement, using the four-probe method, considers the thickness and shape of the films and was calculated as follows:

(6) $$Resistivity\;\left(\rho \right)=(V/I)2\pi S$$

where S is the distance of the tips, V is the measured voltage, and I is the current applied.

Finally, the electrical conductivity (σ) was calculated as follows:

(7) $$\sigma =\frac{1}{\rho }$$

### 2.15. Photoluminescence

The photoluminescence technique was used to investigate the optical properties of the films. The films were excited by a 375 nm CW laser (CNI, MDL-III-375). A 45° focused laser beam was directed at the sample surface, and perpendicular photoluminescence spectra were obtained using an Ocean Optics HR4000 spectrometer, Dunedin, FL, USA.

## 3. Results

### 3.1. UV–Vis Analysis

**Figure 1:**
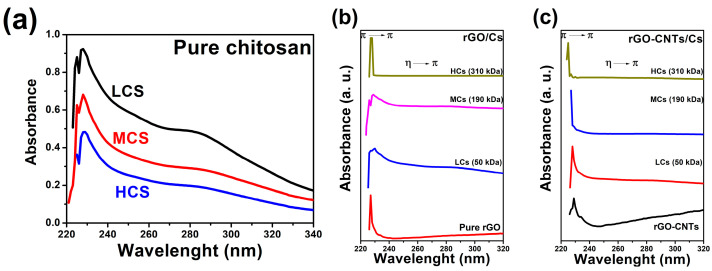
UV–vis absorption spectra of (**a**) pure chitosan, (**b**) rGO/chitosan and (**c**) rGO-CNTs/chitosan solutions from 50, 190, and 310 kDa of molecular weight.

To characterize the absorption features of homogeneous dispersions, the solubilized rGO/Cs and rGO-CNTs/CS solutions were subjected to UV–vis spectroscopic analysis in acetic acid (0.001 mg mL^−1^) in the 200–900 nm wavelength range. Figure 1a depicts UV–vis absorption for pure chitosan of low, medium, and high molecular weight. Spectra reveal the absorption peaks at 228 nm and 280 nm corresponding to the η → π and π → π transitions, respectively. Figure 1b exhibits the absorption spectra of pure rGO and rGO/Cs solutions from low-, medium-, and high-molecular-weight chitosan. The typical electronic transitions related to rGO were observed in the UV–vis spectrum with a maximum absorbance of around 227 nm, which agrees with previous results [14,28,29]. Mixtures of rGO/Cs from low and medium molecular weights exhibit the absorption bands of both the CS and rGO; however, the sample containing reduced graphene oxide/high-molecular-weight chitosan (rGO/HCs) solution shows an intense absorption at around 228 nm, which masks the CS transitions. This high absorption can be attributed to the absorption capacity of chitosan chromophores in the blue region than rGO. Figure 1c shows the UV–vis absorption of rGO-CNTs/Cs solution. We observe that this sample displays a higher capacity to disperse carbon nanotubes due to the repulsion of the charged groups of Cs when dispersed in an acidic medium. The hydrophobic character of rGO/Cs is enhanced by the area of π-conjugated aromatic domains, which facilitates the attachment of CNTs to the rGO/Cs surface [30].

### 3.2. FTIR Spectroscopy

**Figure 2:**
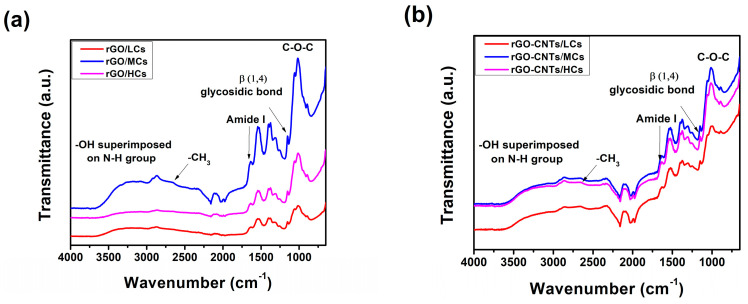
FTIR spectra of (**a**) rGO-Cs and (**b**) rGO-CNTs/Cs films.

The Fourier Transform Infrared (FTIR) spectroscopy proved to be sufficient in analyzing the functional groups and structural modifications of films. rGO/Cs films FTIR spectra are depicted in Figure 2a. The typical superimposed -OH and NH_2_ groups in chitosan are observed. In both reduced graphene oxide/medium-molecular-weight chitosan (rGO/MCs) and reduced graphene oxide/high-molecular-weight chitosan (rGO/HCs), the high wavenumber of 2883 and 2866 cm^−1^ was observed for the antisymmetric and symmetric stretching vibrations of the methyl (-CH_3_) group. Notably, the FTIR spectra of the rGO/LCs film did not exhibit the methyl group peaks, which suggests an interaction between rGO and Cs. The peak related to the acetyl units exhibiting stretching vibration of C=O has undergone a shift toward lower wavenumber values (1640 cm^−1^). A shift toward 1537 cm^−1^ of the protonated amide group’s N-H bending vibration on the positively charged Cs suggests that it interacts with the oxygen-containing groups of the rGO surface. The β-1,4-glycosidic bond of Cs exhibited a shift toward a lower wavenumber (1066 cm^−1^) while the C-O-C stretching vibration shifted from 1091 to 1066 cm^−1^, maybe due to the interaction between -OH groups of Cs and the hexagonal lattice of rGO. Among all the films, the rGO/MCs films exhibited the most intense bands in the FTIR spectrum. The peak at 1637 cm^−1^ is attributed to the -C=C- asymmetric stretching of the rGO. Also, the peaks observed at 3391, 3060, 1642, 1433, and 1091 cm^−1^ correspond to C-OH, CH stretching, -C=C- stretching, CH bending, and C-O stretching, respectively. The centered band at 1637 cm^−1^ could be ascribed to the conjugation of -C=C- with a carbonyl group, displaying reduced intensities [31].

The rGO-CNTs/Cs films FTIR spectra are depicted in Figure 2b. The typical -OH superimposed NH_2_ of chitosan is observed. Antisymmetric and symmetric stretching vibrations of the methyl (-CH_3_) did not appear in the spectra, indicating that the rGO-CNTs and Cs could interact. The peak associated with the acetyl units with stretching vibration of C=O shifted to 1632 cm^−1^. The N-H bending vibration of the protonated amide group of N-H slightly shifted to 1543 cm^−1^, implying that positively Cs interacts with the oxygen groups on the rGO-CNTs surface. β-1,4-glycosydic bond of chitosan shifted to 1151 cm^−1^ and the C-O-C stretching vibration shifted from 1091 to 1057 cm^−1^. All rGO-CNTs/Cs films displayed almost the same intensity in the FTIR, indicating that, by embedding a mixture of CNTs and rGO into Cs, a highly condensed structure is formed. Typical vibrations of carbon-based materials also were observed. The probable interaction between CNTs and rGO is ascribed to the peak at 1637 cm^−1^, which is attributed to the -C=C- asymmetric stretching. The peaks at 3391, 1433, and 1091 cm^−1^ are ascribed to C-OH, -C=C- stretching, and C-O stretching, respectively. The low-intensity band observed at 1637 cm^−1^ can be attributed to the conjugation of -C=C- with a carbonyl group [32]. The interaction of -OH from Cs within rGO-CNTs rings is associated with the stretching of C-O-C at 1070–1010 cm^−1^.

The bands attributed to the amine groups in chitosan appear more intense in reduced graphene oxide–carbon nanotubes/medium-molecular-weight chitosan (rGO-CNTs/MCs) and reduced graphene oxide–carbon nanotubes/high-molecular-weight chitosan (rGO-CNTs/HCs) films. This suggests that positively charged Cs and oxygen-containing groups of carbon-based materials could effectively interact among them. Extensive hydrogen bonding has become broader in reduced graphene oxide–carbon nanotubes/low-molecular-weight chitosan (rGO-CNTs/LCs) films in suitable agreement with previous reports [33]; both -OH and NH^+^ groups could contribute to the ability of chitosan to effectively wrap along CNTs [34] and to interact with the oxygen-containing groups of the rGO. After incorporating CNTs and rGO, the peaks attributed to β (1,4) glycosidic bonds and CH_3_ angular deformation of Cs appear more intense in rGO-CNTs/LCs films, suggesting that the skeleton of chitosan prevails after the addition of carbon-based materials. By increasing the chitosan long chain, a more condensed structure is observed. The slight variations in the stretching of C-CH_3_ groups could be attributed to the π-bonds of chitosan and carbon-based materials. Embedding rGO and rGO-CNTs to chitosan influences hydrogen bonds that contribute to the interactions within the entire system. Interestingly, the antisymmetric and symmetric -CH_3_ groups in chitosan did not appear in rGO-CNTs/Cs films, suggesting that chitosan could interact with the rGO-CNTs structure due to the formation of platelet-like structures observed in SEM micrographs elsewhere in this paper. Chitosan interacting with carbon-based materials could decrease the availability of protonated group concentration in Cs, leading to the formation of condensed structures that have great implications for the optical, mechanical, and electrical properties of films. The synthesis of CNTs and rGO could incorporate oxygen functionalities that could fall under the classification of structural defects, such as edges and basal planes, responsible for lattice vacancies that promote the synergistic effect between carbon-based materials and Cs.

### 3.3. Films Thickness

The thickness of the films is presented in Table 1. From our results, we report that rGO/Cs film thickness ranged around ~50–70 μm, and the rGO-CNTs/Cs thickness films ranged from ~70 to 110 μm. However, the rGO addition increases the thickness of films as chitosan molecular weight also increases, but the incorporation of rGO-CNTs complex decreases the thickness of film as the chitosan molecular weight increases, which agrees with previous reports. A thicker film was formed by adding CNTs and rGO into chitosan, which suggests that carbon-based materials could interact with the long chains of the polymer, reducing the interplanar distance and approaching crystalline structures, as was detailed in the FTIR analysis. Aryaei et al. reported that the modification of the mechanical properties of polymeric films is feasible through distribution, filler content, and orientation, which affects the stress transfer between the polymeric matrix and the filler. Their findings of dried uniform thin films demonstrate that the chitosan/MWCNT composite displayed an average thickness of 0.06 mm and 60 mm [35]. According to SEM images, the rGO-CNTs/Cs films showed more homogeneous and condensed films. It is suggested that thicker films arise from hydrogen bond formation between oxygen-containing groups of rGO and the NH_3_^+^ of chitosan, leading to a more compact structure.

### 3.4. Color and Opacity

The film color values are shown in Table 1. The brightness of the film is indicated by the L* value. Attribute a* denotes greenness/redness, while b* represents blueness/yellowness in the test films. Based on the analysis of the L* parameter, it is evident that rGO has played a role in the decrease in opacity as chitosan molecular weight increases, leading to a decrease in the luminosity of the films. This increase in opacity is attributed to a possible interaction between the oxygen-containing groups or rGO and the positive Cs, which leads to a reduction in the intrinsic yellow color of the pure chitosan. The rGO films appear darker due to their higher L-values, showing less browning. Similar values of *L*-attribute were shown in rGO-CNTs/Cs films. Since film transparency depends on the crystallinity of the polymeric lattice, the correlation between XRD and color results revealed that an increase in crystallinity leads to a decrease in transparency (or an increase in opacity) of the polymer due to a corresponding increase in density. The addition of rGO and rGO-CNTs was found to increase the crystallite size of films. At this point, it is important to mention that non-functionalized CNTs display a high aspect ratio and strong aggregation due to the van der Waals attraction, which leads to the formation of complex networks that contribute to the crystallite size increase [36].

### 3.5. Ion Exchange Capacity and Swelling Degree

Ion exchange capacity (IEC) is a parameter that quantifies the capability of functional groups to bind protons in an aqueous solution, serving as an indirect indication of the proton conductivity of polymers. This parameter is correlated with the amount of interchangeable ionic groups that impact the water absorption properties. The IEC values are shown in Table 2. In pure chitosan film, the ionic groups that provide support to the ion exchangers are -NH^3+^. An increased proportion of protonated groups leads to a rise in the IEC. The role of water content in films is crucial for ionic conductivity, as water molecules are involved in OH- transportation. The high water absorption capacity in the films is a result of the abundant hydroxyl and amino groups of chitosan. The increase in the percentage of swelling degree (DS) in films resulted in high IEC values. Nhung et al. reported that the increase in water absorption is predominantly attributed to the increase in the IEC value. The highly hydrophilic polar groups in Cs are responsible for the increase in swelling ratio and IEC values, thereby augmenting the water absorption ability of films. The transfer of ions relies on the absorption of water. An increased uptake of water could lead to better ionic conductivity [37]. IEC values in rGO/Cs films dropped because of scarce functional groups able to transport electrons. Positively chitosan and highly charged density of oxygen-containing groups in rGO could promote the formation of the condensed structure of films from LCs to HCs molecular weight via hydrogen bonds. Interestingly, the IEC values in rGO-CNTs/Cs films remain almost constant as chitosan molecular weight increases attributed to the highly condensed structures.

The swelling of chitosan can be influenced by chemical composition (i.e., chain flexibility, molecular weight, chemical cross-linking, and solvent effect) and the porous structure of the polymer. The highest percentage of swelling is referred to as a more rigid and condensed structure as chitosan molecular weight increases (Table 2).

The results of this research are consistent with the typical values of three-dimensional polymeric networks, which are appropriate for swelling without disrupting their structure for up to 48 h, at which point absorption equilibrium was established [38]. The rGO/Cs and rGO-CNTs/Cs films exhibited a decrease in the percentage of swelling degree, maybe due to the formation of a more condensed structure, which leads to reduced swelling. The addition of rGO and rGO-CNTs into Cs led to a less hydrophilic network structure, primarily due to functional groups like amide, amine, hydroxyl, and a non-porous network. Carbon-based materials can influence the crystalline structure of chitosan, diminishing the availability of hydroxyl groups. Cohesive forces are involved in CNTs and rGO assembly, resulting in enhanced mechanical properties and reduced water swelling. The FTIR spectrum of films showed a less intense OH band, which could suggest the interaction between positive Cs and oxygen-containing groups of CNTs and rGO. This interaction causes a decrease in the accessibility of polar groups, leading to a reduction in the percentage of DS.

**Figure 3:**
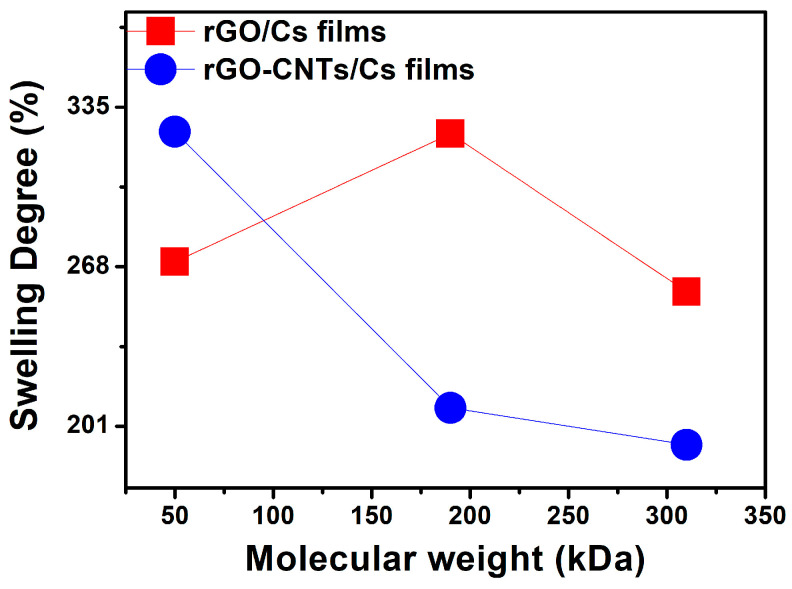
Swelling degree percentage of rGO/Cs and rGO-CNTs/Cs films.

Chitosan exhibits hydrophilicity, and the rapid diffusion of water into its structure results in the swelling of films prior to their degradation. During the initial stages of the hydration process, bond breaking occurs prior to chitosan degradation. Nevertheless, swelling suppresses degradation. When an equilibrium state is reached due to the presence of salts, residual acetic acid, and other factors, the swelling profile of chitosan films reaches a peak and thereafter decreases with time. The influence of rGO on the decrease in DS of CS-rGO films is related to the presence of hydrophilic groups, cross-linking, and polar ionic groups present in the chitosan structure. This allows it to have a high interaction with an ionic solution [38,39]. We suggest that the low DS value observed due to the incorporation of rGO into chitosan may be attributed to a possible repulsion of water molecules by the presence of negative charges on the film surface. The pores of chitosan can be filled with nano-sized graphene, thereby substituting free water without any swelling effect, thereby complicating the rehydration process. This phenomenon may be attributed to the size of rGO nanosheets, which prevent water molecules from entering the chitosan during the rehydration process. Additionally, the material undergoes dehydration, resulting in a decrease in size. This may be accompanied by a rearrangement of the chitosan molecules coating the rGO, thereby limiting the hydration process. From Figure 3, it is observed that the film rGO/MCS shows a maximum DS, attributed to the availability of amino groups interacting with the hydrophilic groups of water. According to the FTIR analysis, the superimposed OH-NH_2_ band is more intense in the rGO/MCS film than in the rGO/LCS and rGO/HCS films. The percentage of DS observed in films containing a mixture of rGO-CNTs exhibits a distinct profile as the molecular weight increases. For all three molecular weights of chitosan with rGO-CNTs, the intensity of the superimposed OH-NH_2_ band was similar. The decrease in DS can be attributed to the presence of CNTs, which confer higher negative charges, which in turn cause higher repulsion of water molecules [40].

### 3.6. XRD Diffraction Analysis

**Figure 4:**
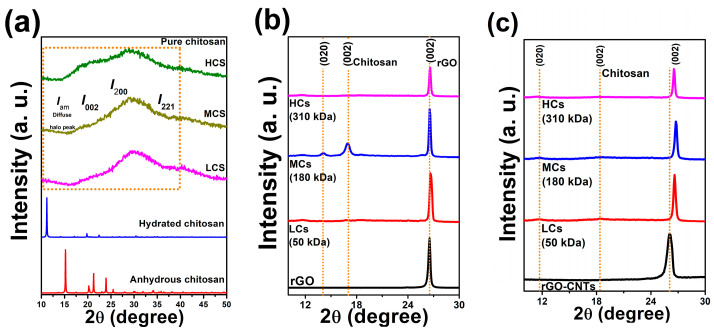
XRD patterns of (**a**) low-, medium-, and high-molecular-weight chitosan, (**b**) rGO/Cs films, and (**c**) rGO-CNTs/Cs films.

The changes in the crystallinity of films were identified using X-ray diffraction analysis. Figure 4a–c display the XRD patterns of Cs (LCs, MCs, and HCs), rGO/Cs, and rGO-CNTs/Cs films. The diffraction peaks observed at around 2θ = 11° and 2θ = 20° are consistent with prior research [41]. The XRD patterns of films exhibit a significant reduction in peak sharpness at 2θ = 10° upon the addition of rGO-CNTs. The reflections at 2θ = 11.61° and 2θ = 16.91° seen in both LCs and HCs chitosan films correspond to form I and form II. As shown in Figure 4c, the rGO-CNTs/Cs film’s XRD pattern exhibits a decrease in the 2θ = 11.5° peak, which is absent in rGO films, indicating significant changes in the Cs form I, as displayed in Table 3.

According to Barbosa et al. [42], the chitosan crystallinity is highly influenced by molecular weight. The XRD patterns have shown that both CNTs and rGO exert a considerable influence on the crystalline structure of Cs, as evidenced in Figure 4b,c. Graphemic structures could influence the crystalline structure of MCs chitosan since the peaks at 14.1° and 16.95° still appear. The incorporation of rGO was found to have a significant impact on the crystalline structure of chitosan, as indicated by the XRD pattern of the rGO/Cs film. The diffractograms of rGO/LCs and rGO/HCs films depict low-intensity XRD peaks of chitosan at 16.95°. The observation suggests that the composite structure formation is significantly influenced by the intercalation between rGO and chitosan, which may be ascribed to the interaction between the oxygen-containing groups of rGO and the Cs amine groups. The peak at 2θ = 26.1° typical of carbon-based materials shifted to higher angles is due to the strain in both inter- and intra-layer directions. FWHM (Full Width at Half Maximum) values from rGO and rGO-CNTs films (002) peaks suggest that, after incorporating carbon materials into LCs and MCs, a more crystalline structure was obtained, demonstrating that carbon-based materials are responsible for the changes in the crystallinity of the films. The method to calculate the CrI is based on the formula showing the relationship between the intensity of a crystalline reflection and the intensity of the minimum peak at 2θ = 12–16°, which conditionally describes the diffuse halo peak (I_am_). Recently, Podgorbunskikh et al. [41] emphasized the applicability of the method for calculating the crystallinity index (CrI) of chitosan. As is well known, to calculate the CrI, the method that shows the ratio between the intensities of the peaks of the crystalline and amorphous region of chitosan, centered at 2θ = 19–20° and 2θ = 12–16°, respectively, is used. However, the applicability of the method decreases substantially if we have a mixture of anhydrous and hydrated polymorphs. The method based on the ratio of the crystalline peak area to the total area of the XRD pattern has been proposed to calculate the crystallinity index of samples consisting of a mixture of polymorphs more accurately. In our work, we analyze the amorphous and crystalline region of films by the deconvolution method, as can be observed in the Supplementary Information (Figure S2): the XRD pattern was deconvoluted into internal peaks (deconvolution into five or six peaks was carried out for a more accurate description) using the Voight approximation. The crystallinity index was calculated from the ratio of the sum of the areas of the crystalline regions and the sum of the areas of the amorphous component peaks.

$$CrI=S_{cryst}/S_{total}$$

For the chitosan used in this work, the reflections corresponding to the hydrated crystalline form were detected at ~2θ = 10° (020) and 20° (200). The single reflection (220) is not observed in the XRD pattern for any of the films fabricated with the three chitosan molecular weights studied; however, by applying the deconvolution method, it was possible to observe the component of this reflection. The peaks corresponding to (100), (200), and (001) reflections in graphite described the crystalline structure of organic materials with a high carbon content, such as CNTs. The occurrence of aromatic rings is confirmed by a symmetrical (002) peak centered at 2θ = 26.1° in the XRD diffractogram of rGO and CNTs-rGO with an interplanar distance of ~3.39 Å. For more information, see the Supplementary Information (Figure S3). The non-appearance of the plane (001) suggests that the reduction process was effective in eliminating oxygen functionalities and restoring the π-π network of the rGO.

### 3.7. Mechanical Properties

Four to six test specimens used to calculate the mechanical properties of films reinforced with rGO and rGO-CNTs were analyzed, and the parameters reported in this work are directly related to the structural cohesivity of the films and imply their resistance to breaking under tension and fracture strain after the film has been broken [43,44]. Geng et al. developed graphene fibers through wet spinning and applied it to the study of interfacial electrolyte complexation by proposing to in situ construct a hierarchical assembly structure composed of well-ordered rGO sheets and chitosan molecules. It has been reported that graphene fibers exhibit a TS value of 875.5 MPa, which is attributed to the robust ionic bonds and hydrogen interactions between rGO and chitosan. Molecular dynamic simulations revealed that a decrease in rGO sheets and Cs molecules was accompanied by the breaking and reforming of interfacial bonds [45]. Our findings demonstrate that the films produced in this work display interesting TS and elongation-at-break (EB) values influenced by the incorporation of rGO and rGO-CNTs into chitosan. The rGO-CNTs/HCs and rGO-CNTs/LCs films showed a maximum and minimum percentage of EB of 8.37% and 2.71%, respectively. rGO-CNTs/Cs films showed improved mechanical performance with enhanced electrical conductivity, giving the films a suitable prospective application. An inadequate dispersion of CNTs is a critical factor in the improvement of mechanical properties due to the formation of nano cracks. As dispersions undergo ultra-homogenization, polymer molecules experience significant mechanical stress due to the simultaneous presence of shear, turbulence, impacts, and cavitation forces [46]. Table 4 summarizes the mechanical performance of the rGO/Cs and rGO-CNTs/Cs films.

Maximum tensile strength of ~83 MPa was achieved by the rGO/LCs films. The mechanical performance of the films shows that the incorporation of rGO and rGO-CNTs into the chitosan significantly influences the tensile strength and Young’s modulus. From the data shown in Table 4, it can be observed that by increasing the molecular weight of the chitosan, the TS of the rGO/Cs films decreases by 60% (rGO/HCs < rGO/MCs < rGO/LCs), while the TS of the rGO-CNTs/Cs films increases by 186% (rGO/HCs > rGO/MCs > rGO/LCs). Although it is important to mention that the SEM micrographs show platelet-like structures in the rGO/Cs films, the rGO-CNTs/MCs films tend to have a maximum stress that allows them to withstand the wind without breaking, perhaps due to the interaction between the rGO and the CNTs, which modifies the crystalline structure and results in a more crystalline structure. The scenario is very similar to Young’s modulus since as the molecular weight of the chitosan increases, the rGO/Cs films show a decrease of 87.5%, while the rGO-CNTs/Cs films show an increase of 16%. The platelet-like structure of the rGO/Cs film could be responsible for the formation of condensed structures that could influence the TS and EB improvement. The dispersion and strong interaction between the chitosan and rGO may result in interfacial stress transfer in the polymer film containing particles, creating a center of stress concentration [47]. The decreased mechanical properties are linked to a loss of rigidity and, in this work, could be attributed to rGO and rGO-CNTs incorporation. The rGO/LCs and rGO/MCs films displayed the highest TS values, 83 and 80 MPa, respectively, but dramatically dropped for rGO/MCs (51 MPa). These TS values could be attributed to a more densely packed structure that is favored by LCs chitosan. On the other hand, the mechanical performance of rGO-CNTs/Cs films is enhanced by the synergistic effect of CNTs and rGO, leading to an increased TS value from LCs to HCs (39, 45, 75 MPa) in suitable agreement with other reports [16,48,49]. More details of the mechanical performance are depicted in the Supplementary Information (Figure S4).

Positively charged chitosan repels each other and is extended in an acidic solution owing to the protonated -NH_3_^+^ groups that lead to wrapping along carbon nanotubes. The -NH_3_^+^ could interact with defected CNT and rGO, inducing a synergistic reinforcement of the physical properties of chitosan. When combined with a suitable TS value, the rGO-CNTs/HCs films displayed the highest EB value, implying a better-quality film. The role of chitosan molecular weight in elongation at break was studied by correlating the -NH_3_^+^ group vibration from the FTIR spectra. We found that if the -NH_3_^+^ group intensity decreases, the elongation at break increases; as the band becomes more intense, the EB value is lower. The highest EB value and the lower intensity of the -NH_3_^+^ group were observed in the rGO-CNTs/HCs film. A synergistic effect of carbon materials into chitosan occurred with an effective interaction between polar groups reacting preferentially in HCs chitosan.

In comparison to pure chitosan films, the toughness value increases with the incorporation of rGO, but not for the films made with rGO-CNTs mixture. This might be due to the wrapping of the chitosan along the surface of the CNTs. In contrast, more cohesive structures are formed when rGO-CNTs are mixed into chitosan. The mechanical properties, such as tensile strength, elongation at break, and elastic modulus, agree with previous reports, thereby enabling films to be utilized in diverse applications. Currently, published studies on chitosan films do not use the synthesis method followed in this research, resulting in variations in the toughness data reported for chitosan films filled with rGO and rGO-CNTs. In addition, there is a scarcity of literature where toughness calculations are performed. Although previously reported toughness values are higher in comparison to chitosan films reported in this work, the methods of synthesis employed by them differ from the one employed in this study [50,51].

### 3.8. X-ray Photoelectron Spectroscopy (XPS)

**Figure 5:**
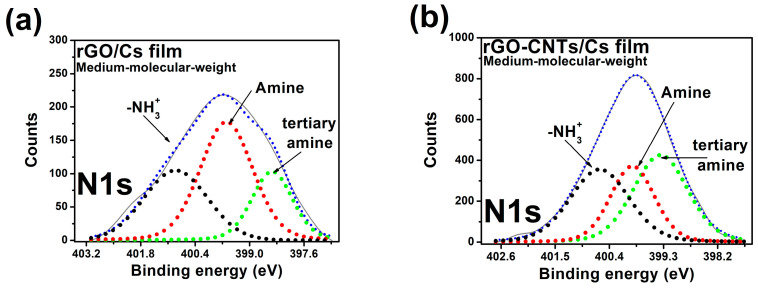
XPS core level N 1s of (**a**) rGO/MCs film and (**b**) rGO-CNTs/MCs film.

XPS measurements allow us to detect O 1s, C 1s, and N 1s in rGO/MCs and rGO-CNTs/MCs films. We settled the photoelectron binding energy (BE) from their corresponding peak positions in the XPS spectra. The binding energy of the C 1s line at 284.5 eV was employed as the reference energy for spectral alignment. XPS spectra films display the changes in the C 1s spectrum; for more information, see Supplementary Information (Figure S5). Figure 5a,b show the core level spectra of N 1s. XPS spectra of the rGO-CNTs/MCs films show the π-π* shake-up feature, which could be thought to restore the *sp*^2^ network of carbon-based materials. The internal components of the N 1s of Cs at 399.4, 400.5, and 401.7 eV of BE were linked to the amine, amide, and protonated amine groups, respectively, according to [52]. The N 1s core level contains three components for rGO hybrid film (Figure 5a), centered at 398.48, 399.60, and 400.87 eV related to the amine and amide groups and -NH_3_^+^, the peak at higher BE is related to the amine groups (Table 5). According to Figure 5b, the N 1s contains three inner components for rGO-CNTs/MCs at 399.3, 399.8, and 400.5 eV, related to tertiary amine, secondary amine, and protonated amine, respectively. Table 6 depicts that the area ratios of A_399_ and A_400_ gradually increase because of the increase in amide groups. The ratio increases with the addition of CNTs and rGO (Table 5). The XPS analysis could support the FTIR results by confirming the interaction between chitosan amine groups and the oxygen functionalities of the rGO. The positively charged chitosan could contribute to electrostatic interaction with the rGO, which is consistent with previous reports [53,54].

### 3.9. SEM Analysis

**Figure 6:**
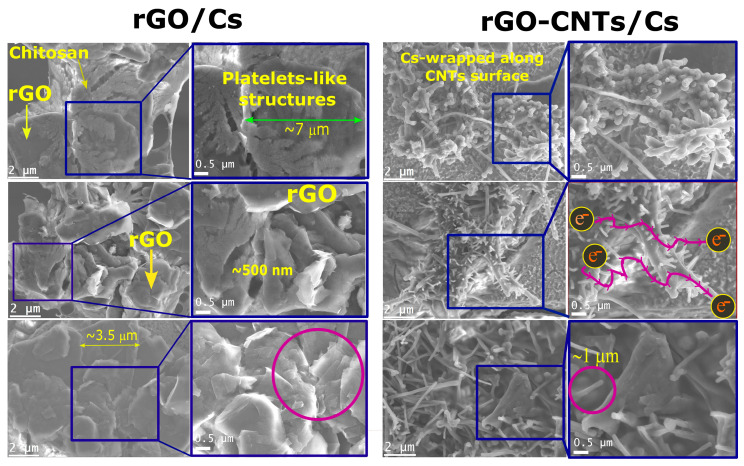
Scanning electron microscopy (SEM) of rGO/Cs and rGO-CNTs/Cs films and zoomed-in sections.

A Scanning Electron Microscope (SEM) was employed to analyze chitosan films with medium molecular weight (rGO/MCs and rGO-CNTs/MCs). The SEM rGO/Cs and rGO-CNTs/Cs images and the zoomed-in section are displayed in Figure 6. The distribution and/or agglomeration level of rGO and rGO-CNTs within the chitosan matrix was confirmed through SEM analysis. According to Lau et al. [55], the micrographs of films exhibit heterogeneous surfaces that consist of protuberances and condensed structures. The aggregation behavior is attributed to the favorable interaction between chitosan and carbon-based materials, as previously mentioned.

Cs bundles observed in SEM images are because of their high polar nature and perhaps responsible for Cs wrapping capabilities. The active polar groups NH_3_^+^ in chitosan can interact with CNTs surface through van der Waals interactions, promoting spontaneous wrapping around CNTs, taking, and helical conformation, leading to the formation of a thin layer on the CNTs surface [56]. The interaction between polymers with inflexible backbones and single-walled carbon nanotubes was investigated by Tallury et al. [57] through molecular dynamics simulations. The wrapping of SWCNTs with polymers having stiff backbones is characterized by more distinct conformations than those with flexible backbones. The decrease in stiffness was observed to be responsible for the reduction in intra-chain coiling. According to Liu et al., the CNTs display a strong tendency to form bundles, which is a key factor in achieving an appropriate homogenization before deposition for the solvent cast. Chitosan interacts with carbon nanotubes, causing them to be randomly distributed within the polymer. According to previous reports, surface decoration led to the complete coverage of carbon nanotube surfaces with chitosan bumps [58]. In this research, the pristine structure of the CNTs was unaffected through a functionalization process. Figure 6 demonstrates the differences between the rGO/MCs and rGO-CNTs/MCs films. Several visible rGO sheets were observed, and a successful blend of reduced graphene oxide with chitosan was used to produce films [16]. It seems that the fabrication method is responsible for the folded layered assembly of rGO/Cs films. SEM images of rGO/Cs films demonstrate that Cs covers the rGO sheets, and the rGO is evenly distributed and forms condensed structures throughout the composite film. The TS and EB values may be explained by the protrusions of chitosan that were formed on the CNTs surfaces and eventually wrapped around the carbon nanotubes, as depicted in Figure 6. We attribute the enhanced flexibility of the films to the better dispersion of carbon-based materials with Cs, and the dispersion behavior of rGO into the Cs matrix could be responsible for the improved mechanical properties.

### 3.10. Resistivity Measurements

A source-measure unit (Keithley Model 4200-SCS system) was employed to measure the resistivity of all films at room temperature. Every measurement was carried out using equidistant tip configurations of 0.040 inches. A gate voltage between −0.001 and +0.001 V was used for a 10 s data collection period. The resistivity (ρ) was calculated from the characteristic I–V curves. Electrical conductivity (σ) was calculated as the inverse of the resistivity (ρ) considering the sample geometry and the thickness, according to Equation (7).

Table 7 displays the electrical conductivity values of the as-prepared films. From previous reports [59], the incorporation of a small quantity of CNTs resulted in a significant enhancement of the film’s electrical conductivity from 1.05 × 10^−5^ to 9.43 × 10^−3^ S/m. The enhanced electrical conductivity could be attributed to the synergistic interaction between CNTs and chitosan, along with dispersion and overlapped structures. According to the SEM images reported elsewhere in this document, it can be concluded that the enhanced electrical characteristics of rGO-CNTs/Cs films are due to the contacts between the tubes and their non-uniform distribution in chitosan, which is an essential factor for ensuring effective transfer of electrical charge. The rGO/LCs and rGO/MCs films displayed conductivity values below 10^−8^ S/cm that decreased for rGO/HCs films. Surprisingly, rGO/Cs films displayed low electrical values in comparison with the rGO-CNT/Cs films, which could be influenced by a strong interaction between the rGO-CNTs complex and chitosan. From SEM micrographs, we observed that rGO and Cs develop platelet-like structures that could hinder the π-π network of rGO, thus decreasing the conductive properties of rGO by diminishing the charge transport along the film due to the strong interaction of the rGO and the chitosan. Thus, conductivity measurements show that increasing the long chain of chitosan diminishes the electrical conductivity, as was observed in the rGO/Cs films as molecular weight increases (1.55 × 10^−9^, 7.45 × 10^−8^, 7.6 × 10^−10^ S/cm). The positively charged chitosan and oxygen functionalities in rGO lead to the formation of a condensed structure with appropriate mechanical performance, but that hinders the charge transport along the film.

The rGO-CNTs do not contribute to the charge transport due to the condensed structures and the strong interaction between rGO-CNTs and chitosan. Yet, CNTs and rGO are highly conductive materials, and the formation of condensed structures rGO-CNTs/Cs seems to decrease in electrical conductivity values as the molecular weight of chitosan increases (1.1 × 10^−4^, 1 × 10^−9^, 1.03 × 10^−9^ S/cm). Interestingly, the rGO-CNTs/LCs film displayed an increase in electrical conductivity (1.1 × 10^−4^ S/cm) among all the films analyzed in this study. An investigation into the formation of electrospray microcapsules was conducted by Gómez et al. [60], who analyzed the effects of chitosan’s molecular weight, ranging from 25 to 300 kDa. The study evaluated the viscosity, surface tension, and electrical conductivity of solutions. It is important to emphasize that the conductivity values of chitosan were collected in liquid solutions, which improved the mobility of charges, and the conductivity values of chitosan were reported to be around 6–7 mS/cm. The conductivity was increased until viscosity delayed charge mobility. A decrease in the viscosity of the solutions at low acetic acid concentrations was associated with a maximum at higher concentrations. Higher concentrations of LCs were associated with a decrease in the viscosity of the solutions at low acetic acid concentrations. The concentration of charges and their mobility, which are critical to the conductivity of the films, are both significantly influenced by the chitosan long chain. As it increases, so does the protonated species. From our findings, we report that *IEC* values increase as Cs molecular weight increases for rGO-CNTs/Cs films, but the rGO/Cs films dramatically dropped the *IEC* values, maybe attributed to the highly condensed structures. The interaction between Cs and rGO may be attributed to the availability of NH_3_^+^ groups and active defect sites in rGO, such as hydroxyl or epoxide groups. These groups promote the adsorption of NH_3_^+^ groups through OH–N and O–HN hydrogen bonds. This leads to an enhanced charge transfer toward hydrogen abstraction from absorbed NH_3_^+^ groups toward the epoxide rings and, consequently, to an enhanced charge transfer to the rGO [61], inducing a more cohesive structure as observed in the SEM analysis, which may be responsible for the *IEC* and electrical conductivity values reported in this paper. The latter may be attributed to the dispersion of rGO, which can be effectively improved by chitosan chains. In the study of Oliva et al. [62] analyzed the adsorption of NH_3_^+^ on CNTs using QM/MM calculations. Their work aimed to investigate the effect of structural defects, such as pentagon–pentagon pairs or Stone–Wales (SW) defects, on NH_3_^+^ adsorption. According to the findings, the NH^3+^ molecules were physisorbed onto SW defects and could form long-range bonds approximately 3 Å from nanotube surfaces. In contrast, the same molecules were chemisorbed onto a vacancy defect by covalent bonding at 1.55 Å. This is because the NH_3_^+^ group can bind to CNTs with either short-range or long-range interactions, according to the defect type on the surface. The features have significant implications for the properties displayed by the rGO/Cs and rGO-CNTs/Cs films discussed in this paper. Cs, with their distinct hydrophilicity due to a high proportion of amino and hydroxyl groups, exhibit a high degree of polymerization that plays a crucial role in the structural arrangement of the modified chitosan [63] can interact with rGO and CNTs through defect sites, which would explain the crystalline arrangement from XRD patterns, which in turn is responsible for the enhanced electrical [64] and PL properties. It is important to consider that non-functionalized CNTs were used in this study. However, vacancies or defects may be intrinsically introduced into CNTs during the fabrication process, enabling NH_3_^+^ molecules to adsorb onto the surface, leading to a restructuring of the CNTs framework [65].

Dynamic molecular simulations have shown that extensive wrapping of chitosan can significantly improve the dispersion and solubility behavior of CNTs. This is possible by utilizing the emulsifying capacity of Cs, which is attributed to its peculiar solubility behavior. Oxygen-containing groups serve as acceptors, enhancing conductivity and behaving as a *p*-type semiconductor [66]. The electrical conductivity of Cs is significantly influenced by its molecular weight attributed to the increased steric hindrance resulting from a higher degree of polymerization, which may lead to interchain interactions and hinder the acceptance of electrons by *p*-type carbon-based materials, eventually provoking a diminution in electrical conductivity. This outcome indicates that increased conductivity is counteracted by chemically bonded carbon-based materials and Cs. Considering the behavior of electrical conductivity in this study about previous reports, we conjecture that oxygen molecules act as acceptors in the *p*-type rGO and rGO-CNTs, increasing the conductivity after the interaction with Cs by adsorbing onto the basal plane due to positively charged molecules of the polymer, allowing to the oxygen-containing groups adsorbed at the defect site acting as a *p*-type semiconductor. Due to the intercalation of the polymer molecules within the graphene layers, the platelets would provide sufficient mechanical and functional performance to polymer matrices. According to Shi et al. [67], graphene is the most used filler for polymers due to its high surface area and exceptional mechanical and electrical conductivity. The rGO and Cs interface is crucial in determining film structure and properties. Under-loading a weak interface can lead to stress concentration and platelet pulling out, which is a severe defect. Surface modification can lead to the desired interface strength for high-performance composites. Chitosan helps to build up a strong interface, enhancing interfacial interactions that provide desired physical–chemical properties. The activity of organic molecules and experimental parameters, including solvent, temperature, and polymer molecular weight, may impact the chemisorption of Cs with carbon atoms on the basal plane of graphene layers, as well as the non-covalent attachment of Cs to the CNTs basal plane through a defect-site carbon nanotube.

### 3.11. Photoluminescence Measurement (PL)

**Figure 7:**
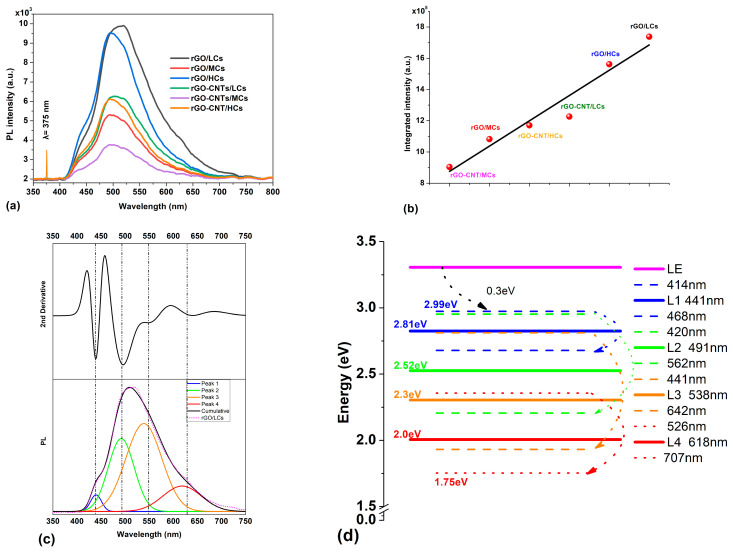
(**a**) PL spectra of rGO/(LCs, MCs, HCs) and rGO-CNTs/(LCs, MCs, HCs) films. For comparison, the laser emission line is shown. (**b**) integrated PL intensity (**c**) PL deconvolution of sample rGO/LCs. (**d**) Energy level diagram depicting the center positions, beginning, and end of the four observed luminescence bands.

Figure 7a displays the PL emission spectra of rGO/Cs and rGO-CNTs/Cs films, both excited about a wavelength of 375 nm. For comparison, the laser emission is shown. Samples exhibit a broadband luminescence covering almost the whole visible spectral range. The integrated intensity (area under the curve) is presented in Figure 7b, where samples rGO-CNT/MCs and rGO/LCs exhibit both the lowest and the highest emissions, respectively. For a better understanding of their spectral composition, Luminescence spectra were deconvoluted. As an example, the PL spectrum of the rGO/LCs sample (Figure 7c) was deconvoluted into four emission bands centered at 441 nm (2.81 eV), 491 nm (2.52 eV), 538 nm (2.3 eV), and 618 nm (2.0 eV), respectively. Both the number and the center position of the deconvolution peaks were carefully selected, considering the second derivative minima of the measured luminescence (see Supplementary Information, Figure S6). Furthermore, during the deconvolution process, the center position, width, and height of deconvolution peaks varied in such a way that the simulated curve best fits with the experimental data, thus maximizing the R square adjusting coefficient. As an attempt to explain the observed luminescence, an energy level diagram is presented in Figure 7d. The diagram depicts the center positions, beginning, and ending of the observed luminescence bands. After 375 nm excitation, the electrons are pumped to the LE level (3.3 eV). However, the results show that the luminescence starts at 414 nm (2.99 eV). This indicates that electrons lose ~0.31 eV energy through non-radiative transitions before they start to decay radiatively to the ground state. Furthermore, the fact that no emissions were detected in the IR region suggests that there are no radiative transitions between the observed luminescence bands, in which case the emissions would be in the IR region (λ > 707 nm; E < 1.75 eV). This suggests that once in LE, electrons lose energy via non-radiative (phonon) transitions to reach energy levels corresponding to bands centered at L1, L2, L3, and L4. To reach these levels, multiphonon transitions (two or three) with energies Eph~0.4 eV should occur, leading the electrons from LE to the observed emission levels. It is known that multiphonon transitions are less likely, so phonons with higher energy Eph ~0.4 eV (2700 cm^−1^ to 3800 cm^−1^) are most likely to reduce the energy of the electrons and populate the energy levels from which the electrons radiatively decay toward the ground state. In the case of lower-energy phonons (0.2–0.1 eV), at least 7–8 phonons would be required for the same transition to occur, which is highly unlikely.

The CIE-1931 chromaticity diagram was produced from the PL spectra data. The chromaticity coordinates of the films demonstrated a gradual shift toward the white region, and the purity of color was calculated (see Figure S7 for more details in the Supplementary Materials section). We found that rGO/Cs and rGO-CNTs/Cs chromaticity values are placed near the locus point in the CIE diagram. Incorporating CNTs and rGO into the chitosan matrix provokes a shift of the wavelength for maximum emission toward the white region of the spectrum in almost all the films (excluding rGO/LCs and rGO/HCs).

Furthermore, the fact that no emissions were detected in the IR region suggests that there are no radiative transitions between the observed luminescence bands, in which case the emissions would be in the IR region (λ > 800 nm; E < 1.55 eV). This suggests that once in LE, electrons lose energy via non-radiative (phonon) transitions to reach energy levels corresponding to bands centered on L1, L2, and L3. To reach these levels, multiphonon transitions (two or three) with energies E_ph_ ~0.4 eV should occur, leading the electrons from LE to the observed emission levels. It is known that multiphonon transitions are less likely, so phonons with higher energy E_ph_ ~0.4 eV (2700 cm^−1^ to 3800 cm^−1^) are most likely to reduce the energy of the electrons and populate the energy levels from which the electrons decay radiatively toward the ground. In the case of lower-energy phonons (0.2–0.1 eV), at least 7–8 phonons would be required for the same transition to occur, which is highly unlikely.

From reference [68], carbon nanotubes can be identified as luminescent centers. From our findings, the films induced the emission of green and white light, which is attributed to an abundance of oxygen atoms and acetamide groups that are responsible for the luminescence of natural polymers such as chitosan [69]. These atoms act as luminescent centers, emitting light due to the high electron density in oxygen-containing groups. The green emission observed between 500 and 525 nm could be due either to donor–acceptor recombination or to a transition from the conduction band to the oxygen antisites, according to Sedky et al. [70]. Several types of defects, including antisite defects and Stone–Wales (SW) defects, can occur within a unit cell. Topological defects, such as the pentagon–heptagon defects generated by the CNTs and rGO processing methods, can create energy levels within the band gap due to the small amount of lattice strain. The defects in graphene and CNTs can be responsible for the observed emission in our research (504–520 nm), causing a shift in the wavelength toward higher values. Amplification of the photoluminescence phenomenon was due to the presence of free electrons in CNTs, which prevented oxygen vacancy defects and promoted the radiative emission mechanism. Kishi et al. reported that the emission of PL is predominantly from the inner walls of carbon nanotubes. The optical and electrical properties of CNTs may be attributed to the interlayer interaction between the inner and outer walls of the material. As a perfect π-conjugated single sheet, graphene lacks electronic band gaps, and it is not photoluminescent; therefore, the creation of energy bandgaps by manipulating the π-electronic network to form quantum-confined *sp*^2^ “islands” in a graphene sheet involves the formation of structural defects, which could be responsible for the creation of bandgaps for emissive electronic transitions, but also directly contribute the bright PL emissions of carbon-based materials. The observed PL emission in graphene materials could be originated from created or induced energy bandgaps in a single graphene sheet and defected multi-layer graphene [71]. More details are depicted in the Supplementary Material (Figure S6). However, based on the above, further work is needed to fully understand the effect of oxygen vacancies on the PL spectra of chitosan-based materials, which are strongly influenced by molecular weight. From the FTIR results, it can be concluded that the oxygen-containing groups belonging to Cs contribute to the high-intensity value observed in the spectrum and that the effect of incorporating rGO and/or CNTs modifies the PL properties of chitosan films. Low-molecular-weight chitosan chains apparently interact more effectively with rGO and CNTs, facilitating the transport of charge carriers and thereby enhancing the electrical conductivity properties reported in this work.

## 4. Conclusions

All films were manufactured using a simple solvent-casting procedure. Afterward, all films were analyzed and characterized to disclose the effect of carbon nanotubes and reduced graphene oxide in chitosan from low to high molecular weight. The results demonstrated that filler addition had a significant effect on the physical, optical, and electrical properties of chitosan films. We found that HCs is an appropriate matrix for the enhancement of mechanical properties of films, but it has an impact on the intrinsic electrical properties of carbon-based materials when added to chitosan; the films behave as insulators with electrical conductivity values around 10^−10^ S/cm. Agglomerated reduced graphene oxide sheets and carbon nanotubes that interact with chitosan molecules due to hydrogen-bonded interactions may have contributed to this unpredicted performance, limiting the charge transport. We have demonstrated that the incorporation of carbon nanotubes and reduced graphene oxide influence the crystalline structure of chitosan, while the average crystallite diameter is affected. The rGO-CNTs/Cs films analysis suggests that carbon nanotubes are the structures that promote electron transfer along the hexagonal network. This was observed in the electrical conductivity values. SEM micrographs showed that chitosan was homogeneously wrapped along carbon nanotubes, which could be the key to the enhanced electrical and PL properties.

In contrast, the addition of rGO does not significantly enhance the electrical properties of chitosan, and it appears that there is no contribution of the reduced graphene oxide to the electrical conductivity. This might be related to the platelet-like structures observed in the SEM micrograph. The late could be due to the interaction of oxygen-containing groups in the rGO surface with amine or hydroxyl in the chitosan molecule. A homogeneous dispersion of rGO into the chitosan matrix significantly improved tensile strength, Young’s module, and elongation at break. Transparent and yellow chitosan films turned opaque, highly influenced by the addition of carbon nanotubes and reduced graphene oxide. rGO-CNTs films significantly reduced the L* parameter more than rGO/Cs films, possibly due to the efficient process of carbon nanotubes to absorb incident light, directing it deeper into voids and contributing to high absorbance. Contrarily, the rGO addition makes a less browning film, as was demonstrated by the opacity analysis.

However, PL measurements showed relevant changes in the PL plot, and the CIE system offered a more precise color measurement. Our results strongly suggest that carbon nanotubes and reduced graphene oxide in chitosan films induce white and green light emissions attributed to the oxygen-containing groups, which are considered luminescent centers due to lone pairs electrons. The color attributes of chitosan films are influenced by adding reduced graphene oxide and reduced graphene–carbon nanotube oxide. From CIELAB analysis, we found that rGO/HCs films a redshift. Moreover, rGO/MCs show a lighter film, as was observed in the CIELAB analysis. PL studies revealed emissions in the blue, green, and red regions. These variances are attributed to the nature of the photoluminescence due to the antisite oxygen-containing groups, the excitation wavelength, and the film production method. The results suggest that CNT and rGO hybrids might have the potential to develop electrically and photoluminescent materials. The resultant broad emission from the films and their characteristics are suitable for numerous valuable applications, including those in bioengineering.

## Figures and Tables

**Table 1:** Thickness, opacity, and color attributes of films.

Films	Color Attributes			ΔE	Opacity (A ∙ mm^−1^)	Thickness (µm)
	L*	a*	b*			
rGO/LCs	34.82	−0.07	1.39	62.84	3.31	48
rGO-CNTs/LCs	34.53	0.05	−0.11	63.16	0.85	106
rGO/MCs	38.54	0.08	0.88	59.12	2.4	52
rGO-CNTs/MCs	31.14	0.02	0.23	66.53	1.18	70
rGO/HCs	36.41	0.26	0.94	61.26	1.9	76
rGO-CNTs/HCs	32.88	−0.11	−0.09	64.80	1.04	60

**Table 2:** Swelling, solubility, and IEC values of films.

Films	DS (%)	SW (%)	*IEC* (meq g^−1^)
rGO/LCs	270.1	21.1	0.0077
rGO-CNTs/LCs	324.7	34.6	0.0073
rGO/MCs	323.9	29.1	0.0074
rGO-CNTs/MCs	208.7	21.4	0.0076
rGO/HCs	208.7	21.4	0.0063
rGO-CNTs/HCs	193.2	15.0	0.0076

**Table 3:** Crystallinity index and crystallite size of the rGO/Cs and rGO-CNTs/Cs films.

Cs Molecular Weight	CrI (%)		Crystallite Size (nm)	
	rGO	rGO/CNTs	rGO	rGO/CNTs
LCs	95	94	31.57	42.08
MCs	56	93	42.08	31.58
HCs	92	97	42.08	42.08

**Table 4:** Summary of mechanical performance of rGO/Cs and rGO-CNTs/Cs films.

Films	Tensile Strength (MPa)	Elongation at Break (%)	Young Modulus (MPa)	Modulus of Toughness (MJ/m^3^)
LCs	72.13 ± 20.03	4.75 ± 1.47	3189.59 ± 524.34	2.06 ± 0.99
rGO/LCs	83.76 ± 20.94	4.39 ± 1.16	4241.22 ± 1344.57	1.92 ± 0.80
rGO-CNTs/LCs	39.88 ± 10.35	2.71 ± 0.68	2225.41 ± 292.00	0.66 ± 0.26
MCs	35.75 ± 2.88	4.75 ± 0.86	1620.60 ± 232.64	1.17 ± 0.32
rGO/MCs	80.43 ± 14.31	5.30 ± 1.81	3897.96 ± 920.32	2.58 ± 1.04
rGO-CNTs/MCs	45.77 ± 5.86	6.33 ± 2.68	2405.75 ± 559.05	1.64 ± 0.59
HCs	49.60 ± 1.55	4.02 ± 0.81	2344.19 ± 466.55	1.21 ± 0.65
rGO/HCs	51.02 ± 9.58	5.22 ± 0.65	2447.75 ± 249.02	1.94 ± 0.39
rGO-CNTs/HCs	74.38 ± 13.65	8.57 ± 2.43	3729.1 ± 487.65	4.93 ± 1.96

**Table 5:** XPS core level C 1s and N 1s of rGO/MCs and rGO-CNTs/MCs film.

XPS Core Level	Films	
	rGO/MCs	rGO-CNTs/MCs
C 1s	285.05	284.91
	285.83	286.02
	286.3	287.05
	287.01	288.65
N 1s	398.32	399.37
	399.63	399.93
	400.93	400.60

**Table 6:** C/O and N/O ratios of rGO/MCs and rGO-CNTs/MCs films.

Films	C (%)	O (%)	N (%)	C/O Ratio	C/N Ratio
rGO/MCs	78.2	14.2	6.2	5.50	12.61
rGO-CNTs/MCs	68.1	25.5	5.0	2.67	13.62

**Table 7:** Electrical conductivity values of the rGO/Cs and rGO-CNTs/Cs films.

Films	σ (S/cm)
rGO/LCs	1.55 × 10^−9^
rGO-CNTs/LCs	1.1 × 10^−4^
rGO/MCs	7.45 × 10^−8^
rGO-CNTs/MCs	1 × 10^−9^
rGO/HCs	7.6 × 10^−10^
rGO-CNTs/HCs	1.03 × 10^−9^

## Data Availability

The original contributions presented in the study are included in the article/Supplementary Material. Further inquiries can be directed to the corresponding author/s.

## Supplementary Materials

The following supporting information can be downloaded at https://www.mdpi.com/article/10.3390/polym16131827/s1, Table S1: Labels for films produced by solvent-casting method. Table S2: Color purity percentage calculated from PL curves. Figure S1: FTIR analysis from 3700 to 2700 cm^−1^ region and the second derivative of rGO/Cs and rGO-CNTs/Cs films. Figure S2: Analysis of the XRD pattern of rGO/Cs and rGO-CNTs/Cs films by the deconvolution method using the Voight approximation. Figure S3: Analysis of the (002) plane of rGO/Cs films and rGO-CNTs/Cs films. Figure S4: Mechanical properties of rGO/Cs films and rGO-CNTs/Cs films from LCs to HCs chitosan (a) stress–strain curves, (b) Young’s modulus, (c) tension strength, and (d) elongation-at-break percentages. Figure S5: XPS core-level C 1s of (a) rGO/MCs film, and (b) rGO-CNTs/MCs film. Figure S6: PL spectra deconvolution of the rGO/Cs and rGO-CNTs/Cs films using the multiple Gaussian peak fit. The second derivative of the cumulative peak is added as additional information in relation to the deconvolution process. Results reveal that second derivative minima are in well agreement with the center wavelength of the subpeaks maxima. The R^2^adjusting coefficient was higher than 0.999 for the deconvolution of all samples. Figure S7: CIE diagram extracted from PL data.
