# Supplementary material for: Influence of Reduced Graphene Oxide and Carbon Nanotubes on the Structural, Electrical, and Photoluminescent Properties of Chitosan Films
Source: Polymers (Basel). 2024 Jun 27;16(13):1827. doi: 10.3390/polym16131827 (PMC11243828; doi:10.3390/polym16131827)
Supplement: Supplementary file 1 [file polymers-16-01827-s001.zip › polymers-3060445-supplementary.pdf]

Influence of reduced graphene oxide and carbon nanotubes on the structural, electrical and photoluminescent properties of chitosan films

Jesús R. González-Martínez[1], Ana B. López-Oyama [2,3*], Deyanira Del Ángel-López [2], Crescencio García-Guendulain [4*], Eugenio Rodríguez González [2], Eder U. Pulido-Barragán [2,3], Felipe Barffuson-Domínguez [5], Aurora G. Magallanes-Vallejo [2], P. J. Mogica-Cantú [2].

[1] Departamento de Investigación en Física (DIFUS). Universidad de Sonora. Blvd. Transversal S/N. 83000. Hermosillo, Sonora, México
[2] Centro de Investigación en Ciencia Aplicada y Tecnología Avanzada, Unidad Altamira del Instituto Politécnico Nacional. Km 14.5 Carr. Puerto Industrial. 89600. Altamira, Tamaulipas, México
[3] Conahcyt-Cicata Unidad Altamira IPN. Km. 14.5 Carretera Puerto Industrial. 89600. Altamira, Tamaulipas, México
[4] Tecnologico de Monterrey, Escuela de Ingeniería y Ciencias. Blvd Petrocel Km 1.3. 89603. Altamira, Tamaulipas, México
[5] Departamento de Física. Universidad de Sonora. Blvd. Transversal S/N. 83000. Hermosillo, Sonora, México
* Correspondence: ablopezoy@conahcyt.mx (A. B. L. O); crescencio.garcia@tec.mx (C. G. G.);

Supporting Information Tables and Figures

Table S1: Labels for films produced by solvent-casting method

Chitosan molecular weight (kDa)	Label assigned according to chitosan molecular weight	Label for films produced with reduced graphene oxide / chitosan	Label for films produced with reduced graphene oxide-carbon nanotubes/chitosan
**50**	LCs	rGO/LCs	rGO-CNTs/LCs
**190**	MCs	rGO/MCs	rGO-CNTs/MCs
**310**	HCs	rGO/HCs	rGO-CNTs/HCs

Table S2: Color purity percentage calculated from PL data

Films	Color purity (%)
rGO/ LCs	20.63
rGO-CNTs/ LCs	17.13
rGO/MCs	18.95
rGO-CNTs/MCs	16.34
rGO/HCs	24.92
rGO-CNTs/HCs	19.62

$$Color\ Purity = \frac{\sqrt{(x - x_s)^2 + (y - y_s)^2}}{\sqrt{(x_d - x_s)^2 + (y_d - y_s)^2}} * 100$$

Where:
x and y are the CIE coordinates of the entire spectrum.
x_s and y_s are the CIE coordinates of the standard illuminants of white light.
x_d and y_d are the CIE coordinates of the dominant wavelength.

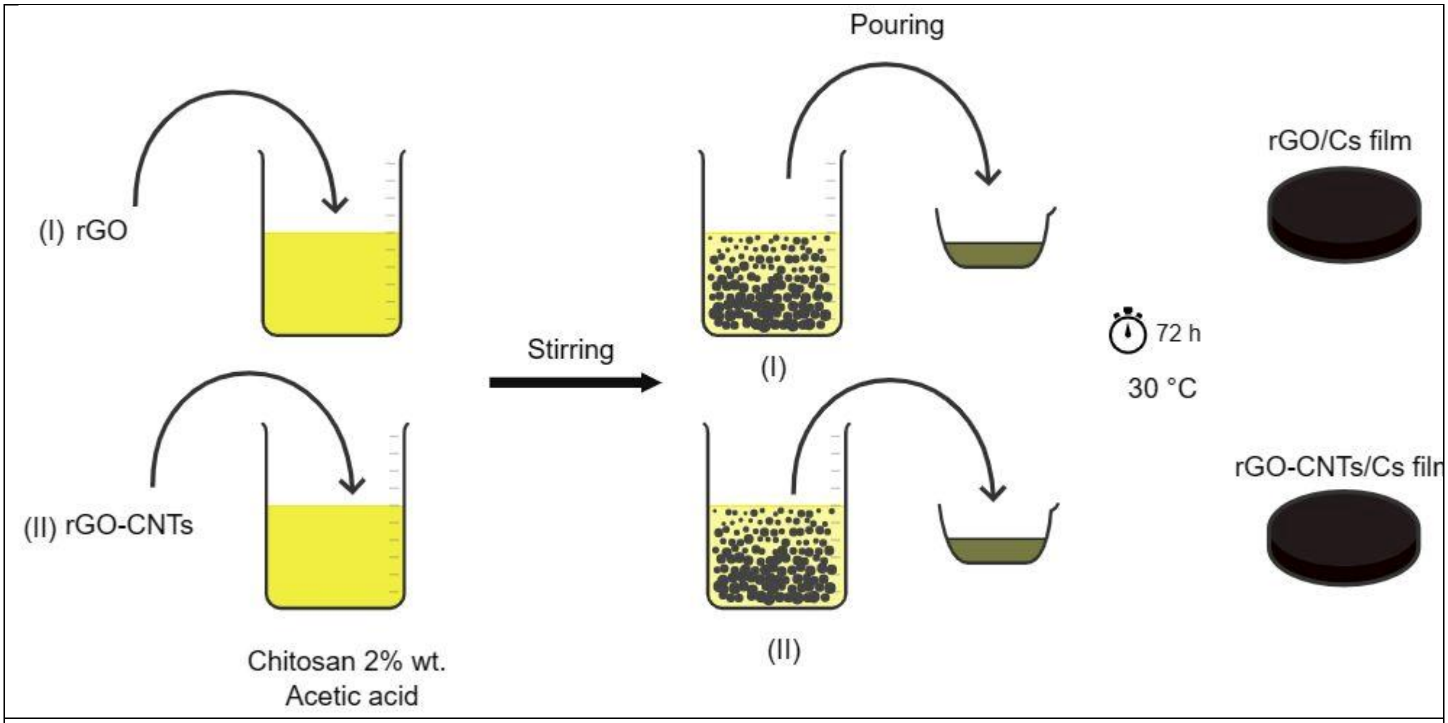

Figure S1. Fabrication of films

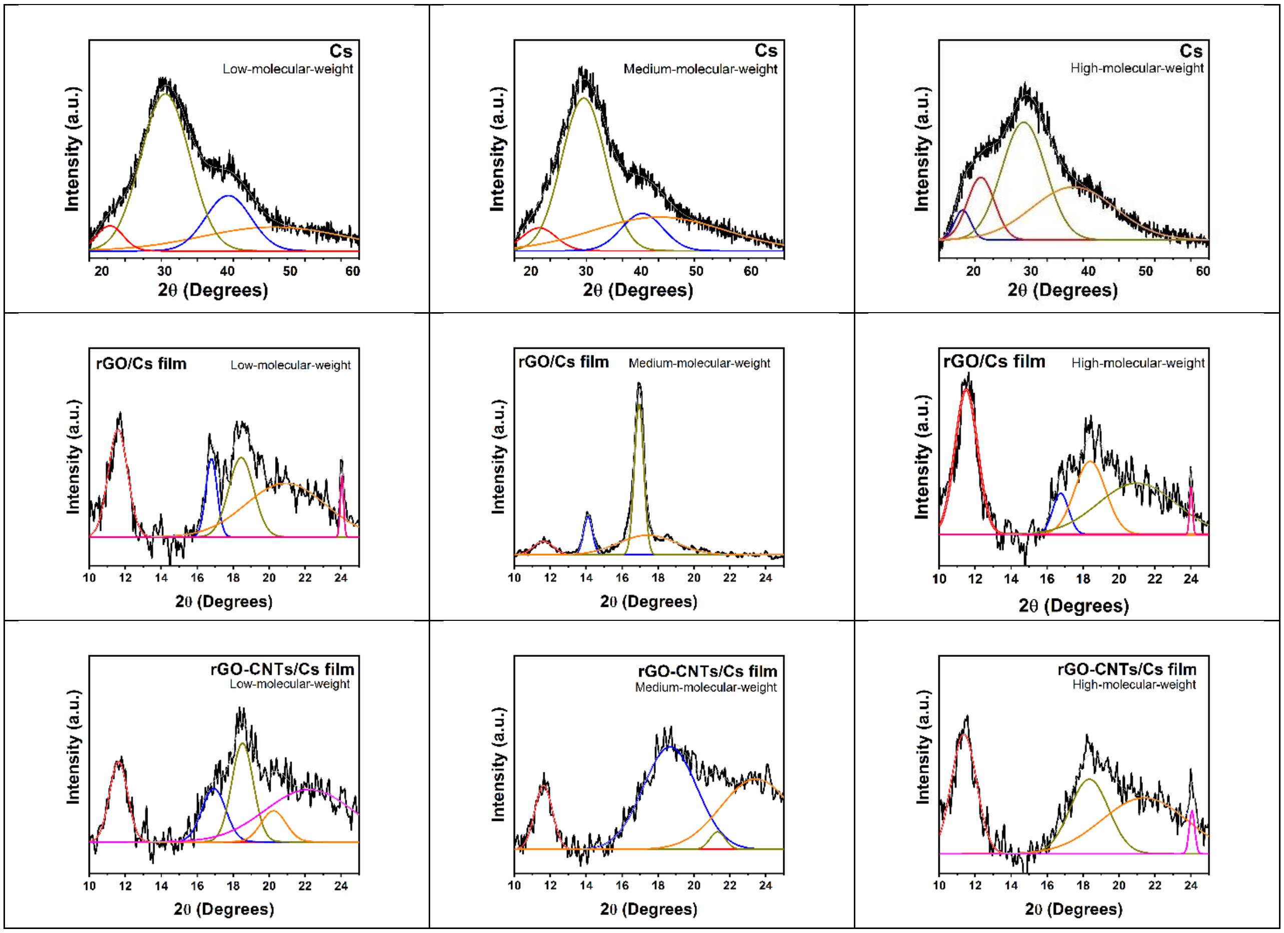

Figure S2: Analysis of the XRD pattern of rGO/Cs and rGO-CNTs/Cs films by the deconvolution method using the Voight approximation

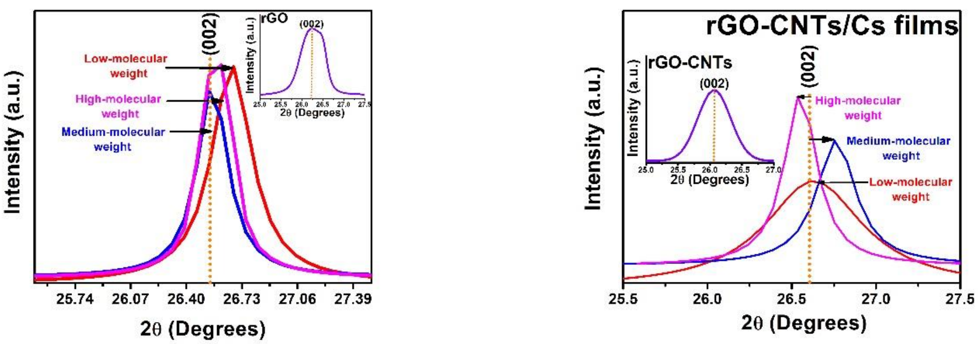

Figure S3. Analysis of the (002) plane of rGO/Cs films and rGO-CNTs/Cs films

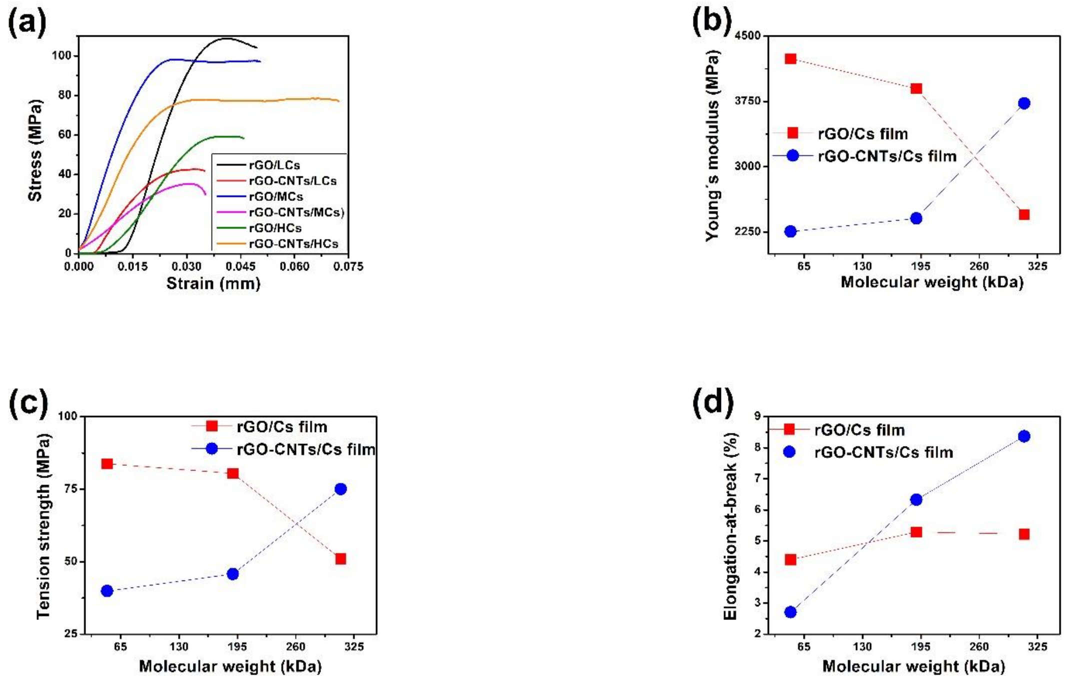

Figure S4. Mechanical properties of rGO/Cs films and rGO-CNTs/Cs films from LCs to HCs chitosan (a) stress-strain curves, (b) Young´s modulus, (c) tension strength and (d) elongation-at break percentages

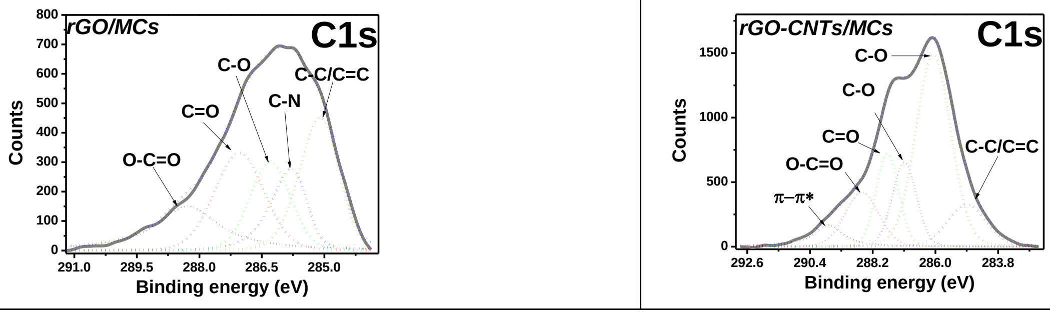

Figure S5. XPS core-level C 1s of (a) rGO/MCs film, and (b) rGO-CNTs/MCs film.

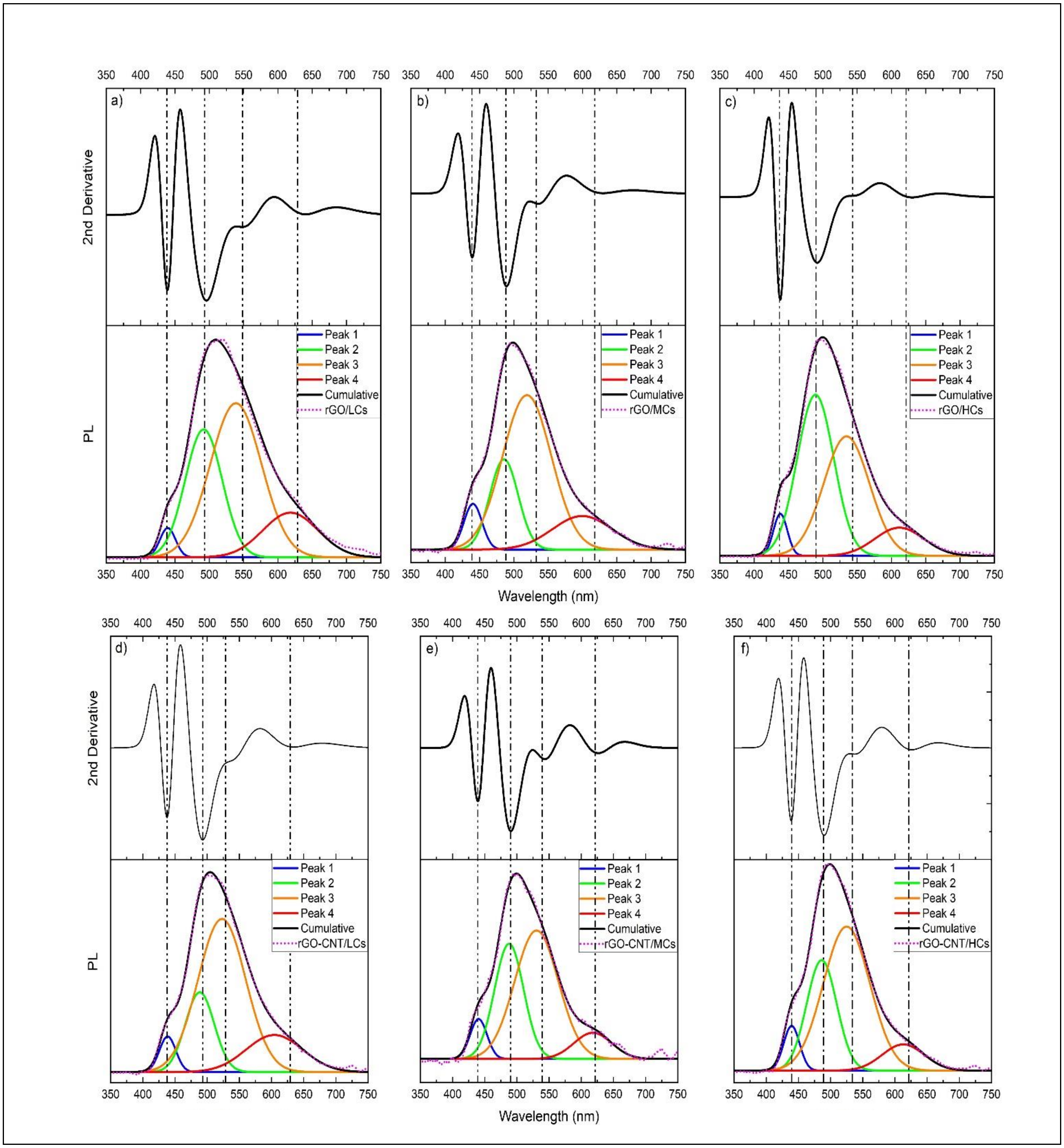

a)
b)
c)
d)
e)
f)
2nd Derivative
PL
Peak 1
Peak 2
Peak 3
Peak 4
Cumulative
rGO/LCs
rGO/MCs
rGO/HCs
rGO-CNT/LCs
rGO-CNT/MCs
rGO-CNT/HCs
Wavelength (nm)
350 400 450 500 550 600 650 700 750

Figure S6. PL spectra deconvolution of the rGO/Cs and rGO-CNTs/Cs films using the multiple Gaussian peak fit. The second derivative of the cumulative peak is added as additional information in relation to the deconvolution process. Results reveal that second derivative minima are in well agreement with the center wavelength of the subpeaks maxima. The R^2adjusting coefficient was higher than 0.999 for the deconvolution of all samples.

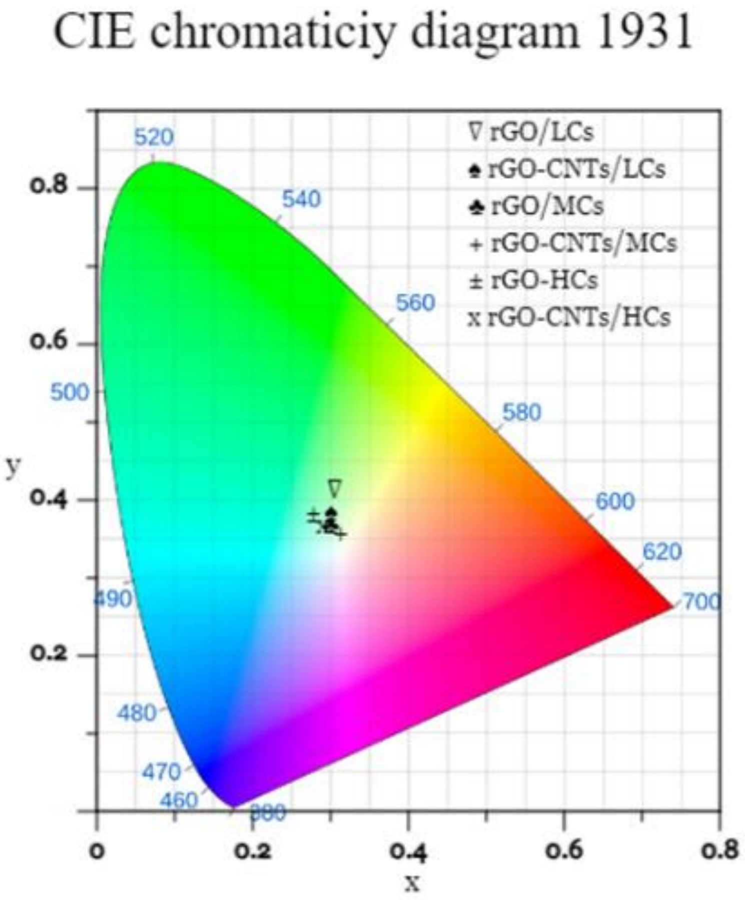

Figure S7. CIE diagram extracted from PL data
